# Correcting for a Baseline Difference in Group Comparisons on a Binary Outcome: Covariate Adjustment or Analysis of Change?

**DOI:** 10.1002/sim.70549

**Published:** 2026-04-16

**Authors:** Gerard J. P. Van Breukelen

**Affiliations:** ^1^ Department of Methodology and Statistics, CAPHRI Care and Public Health Research Institute, Graduate School of Psychology and Neuroscience Maastricht University Maastricht the Netherlands

**Keywords:** binary outcomes, change from baseline, covariate adjustment, Lord's paradox, nonrandomized group comparisons, ordinal regression

## Abstract

Effects of treatments or exposures are evaluated by comparing a treated or exposed group with a control group after treatment or exposure. A baseline group difference can be accounted for by covariate adjustment or by analyzing change from baseline. For quantitative outcomes these two methods can give contradictory results (Lord's paradox) and especially covariate adjustment is questionable for nonrandomized group comparisons. This paper explores analogous methods for the case of a binary outcome, specifically logistic regression of the outcome on group and baseline, ordinal regression of change from baseline on group, and mixed logistic regression and generalized estimating equations (*GEE*) for repeated measures. The methods were compared mathematically, conceptually in terms of causal diagrams and estimands, and numerically on fictitious scenarios that varied in whether groups differed at baseline and/or in change over time on the logodds scale. The methods were also compared on a smoking prevention study among school children. The scenario results were similar to those published for quantitative outcomes: Logistic regression of outcome on group and baseline gave almost the same results as mixed logistic regression and *GEE* without parameter for a baseline group difference. Ordinal regression of change from baseline gave almost the same results as mixed logistic regression and *GEE* allowing for a baseline group difference. These (near‐)equivalences are in line with two mathematical proofs in this paper. Further, in data sets with a baseline group difference, covariate adjustment and change analysis led to contradictory conclusions. The results from the smoking prevention study confirmed the above results and showed Lord's paradox.

## 1 Introduction

In health research, the effect of a treatment or an exposure on a quantitative outcome like forced expiratory volume in pulmonology or total score on Beck's depression inventory in clinical psychology, is usually evaluated by comparing a treated or exposed group with a control group on the outcome measured after treatment or exposure. Unless the study design is a randomized trial, possible group differences at baseline (before treatment or exposure) need to be accounted for. This can be done by either of two methods: Regressing the outcome (posttest) on group and on baseline (pretest) as a covariate or comparing groups on change from baseline (posttest minus pretest). In both cases, adjustment for demographic and other covariates can be made, but this extension will initially be ignored for clarity of exposition. A persistent problem is that the two methods can give contradictory results if the groups differ strongly at baseline. This phenomenon is known as Lord's paradox [1, 2] and has been studied by many authors [3, 4, 5, 6, 7, 8, 9, 10, 11, 12, 13, 14, 15, 16, 17, 18, 19, 20, 21]. In what follows, the terms baseline and outcome will be used, but these can be replaced with pretest and posttest respectively, depending on the reader's domain of interest (e.g., medicine or psychology).

The literature shows that covariate adjustment is preferred to change analysis in randomized studies (because of power) and in studies with treatment assignment depending on the person's baseline (because of bias). These two designs have in common that groups come into existence after the baseline measurement. In contrast, for comparisons of pre‐existing or natural groups, such as a treated community A with an untreated community B, it is not clear when to use which method. Change from baseline analysis has long been known to be equivalent to the group by time interaction test in a repeated measures ANOVA or a mixed linear regression, with baseline and outcome as repeated measures and fixed effects of group, time, and group by time. More recently, it was shown [16, 17, 22] that covariate adjustment is equivalent to the group by time effect test in a constrained mixed model that assumes absence of a group difference at baseline (i.e., coding time as 0 for baseline and 1 for outcome, and leaving the group effect out of the model). This result explains why covariate adjustment is the best method if treatment assignment is by randomization or based on the person's baseline, as there can be no group effect at baseline in these two designs because groups are created after the baseline measurement. But the result also implies that comparisons of natural groups should not in general be based on covariate adjustment, that is, on comparing groups on the outcome adjusted for baseline as covariate. This does not mean that change from baseline analysis is valid, however, as that method makes another strong assumption, specifically that groups would have shown equal change if neither had been treated or exposed.

The above results and publications concern group comparisons on a quantitative outcome, but outcomes are frequently binary in health studies, such as smoking status in health promotion or the presence of some recurrent health problem. Group comparisons on a binary outcome measured before and after treatment or exposure, just like those on a quantitative outcome, can use various statistical methods, specifically logistic regression of outcome on group and baseline (covariate adjustment), ordinal regression of change from baseline on group, and mixed logistic regression and generalized estimating equations (*GEE*) for repeated measures. The aim of this paper is to compare these methods mathematically and by analysis of some fictitious scenarios to find out (a) if Lord's paradox then also occurs, and (b) if the relations between covariate adjustment, change analysis, and mixed regression for repeated measures as shown for quantitative outcomes also hold for binary outcomes. The focus will be on nonrandomized group comparisons, and the reader is referred to [23, 24, 25] for the case of covariate adjustment in randomized trials with a binary outcome. The outline of this paper is as follows. The next section introduces as motivating example a smoking prevention study among primary school children in the Netherlands [26, 27], summarizing its design, analysis, and some results relevant to the present work. Subsequently, the statistical methods for binary outcomes mentioned above are defined and compared mathematically. Next, the methods are evaluated in terms of estimands and causal diagrams [9, 28] for several designs, pointing out when and why each method estimates the so‐called “average causal effect” (ACE, [13]) with or without bias. Thereafter, the data from five hypothetical group comparisons will be presented, differing in the presence or absence of a baseline group difference and in whether the groups show equal change, or converge, or diverge, on the logodds scale. The methods are applied to each data set and their results are compared. Last, the methods are compared on the smoking prevention study. The discussion summarizes all results and their relevance to nonrandomized group comparisons on a binary outcome and points out some limitations of this work and possible future extensions.

## 2 The Smoking Prevention Study

The short‐term results of a cluster randomized trial on smoking prevention among primary school children age between 10 to 13 years from 156 Dutch primary schools were reported in [26]. Schools were randomly assigned to one of four conditions: an existing in‐school social influence program, an out‐of‐school program consisting of tailored letters with smoking prevention messages that had been developed by the authors, a combination of both interventions, and control. The primary outcome was current smoking, measured by self‐report and defined as having smoked in the last month. Measurements were obtained before intervention (T0), 5 months later (T1), and again 4 months thereafter (T2), as well as at three further follow‐ups [27]. In view of massive (i.e., more than 60%) drop‐out at those last three follow‐ups the present work only uses the first three measurements. Further, for simplicity this work compares the out‐of‐school tailored intervention with the control condition, ignoring the in‐school and combined conditions for two reasons. First, the authors of [26] were primarily interested in the out‐of‐school intervention, and second, in spite of the cluster randomization the percentage current smokers at baseline was significantly lower in that condition than in the control condition (for details, see Section 6 of this paper). Finally, the clustering of children in schools will initially be ignored as the methods in this paper are for unclustered data, but the results after accounting for the clustering will also be presented.

**FIGURE 1:**
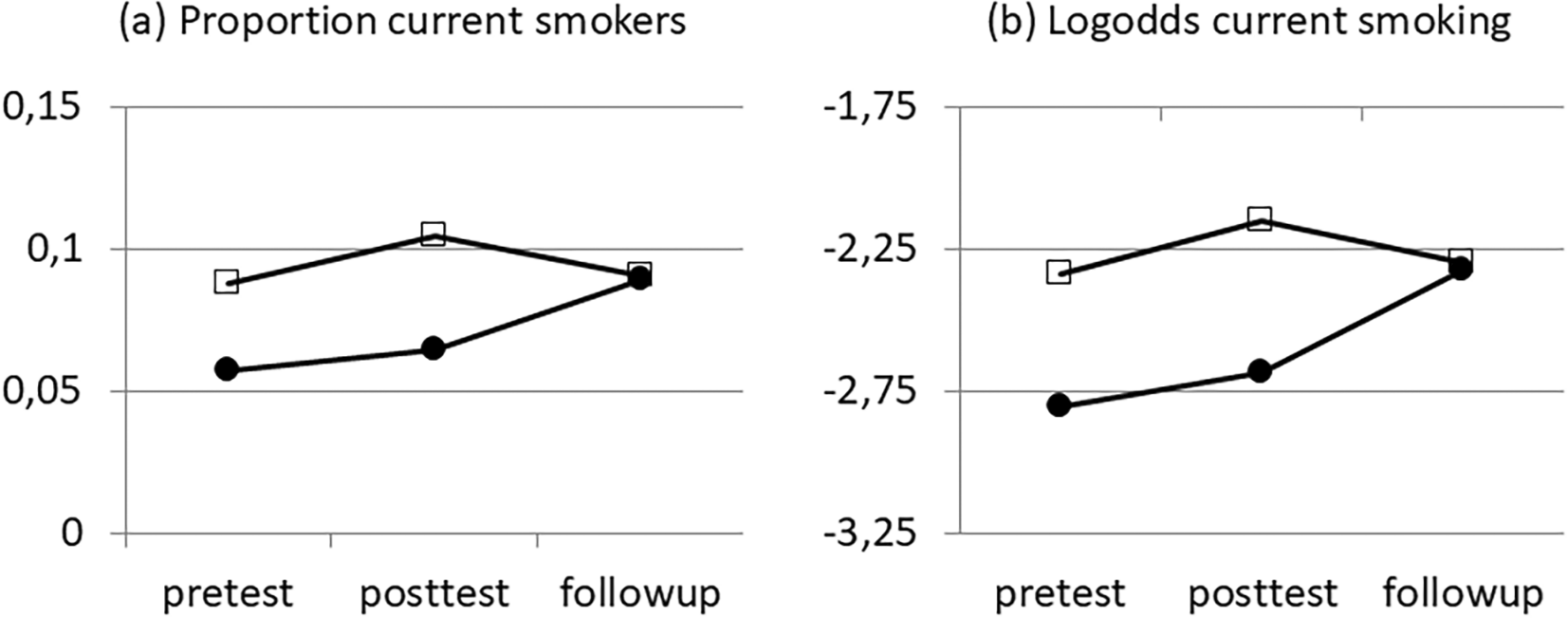
Time course of current smoking per treatment arm (⚫ = tailored letters; □ = control).

Figure 1 shows the time course of the percentage current smokers in both conditions, suggesting parallel change in both conditions from T0 to T1, followed by convergence at T2. Drop‐out from T0 to T1 and T2 was about 10% in both conditions and including versus excluding these drop‐outs into the analysis hardly changed the results of the statistical analyses, which can be summarized as follows (for details, see Section 6): Comparing both conditions at T1, using T0 as covariate, gave a significant intervention effect, whereas comparing both conditions with respect to change from T0 to T1 did not do so at all. In contrast, comparing both conditions at T2, using T0 as covariate, indicated no intervention effect at all, whereas comparing with respect to change from T0 to T2 did. Finally, comparing both methods on T1 and T2 (i.e., T2 as outcome with T1 as covariate versus change from T1 to T2 as outcome) gave the same results as the comparison on T0 and T2. So, Lord's paradox occurred for every pair of time points. Accounting for the clustering of children in schools weakened the paradox for the first two pairs of time points, but not for the last pair.

## 3 Statistical Methods and Their Mathematical Relations

This section presents the statistical methods for the analysis of a group difference on a binary outcome measured before and after treatment or exposure, and compares these mathematically. Section 4 compares the methods in terms of causal diagrams and estimands. Section 5 compares the methods on five fictitious data sets, each with a sample size of 1000 per group to minimize sampling error but differing in the presence of a baseline group difference and of a group difference in change over time. Finally, Section 6 returns to the smoking prevention study, comparing the methods on real data.

### 3.1 Logistic Regression of Outcome on Group and Baseline

Denoting the outcome as *Y*, the baseline as *X*, and group as *G* (0 = control, 1 = treated), the model for analysis is in terms of logodds:

(1) $$\mathrm{LN}\left(\frac{P\left(Y=1\right)}{P\left(Y=0\right)}\right)=\beta_{0}+\beta_{1}G+\beta_{2}X$$

The effect of interest is here $\beta_{1}$, the group outcome difference on the logodds scale, or $\exp\left(\beta_{1}\right)$, the odds ratio, adjusted for baseline.

### 3.2 Ordinal Regression of Change From Baseline on Group

Compute first the change from baseline score as *D* = *Y*−*X*, with *D* for difference, which ranges from −1 (for change from 1 to 0) to +1 (for change from 0 to 1). One popular ordinal regression model is the cumulative logit model ([29], equation (6.4)), which for the present change score is:

(2) $$\mathrm{LN}\left(\frac{P\left(D\geq 1\right)}{P\left(D<1\right)}\right)=\alpha_{0}+\alpha_{1}G\;\text{and}\;\mathrm{LN}\left(\frac{P\left(D\geq 0\right)}{P\left(D<0\right)}\right)=\alpha_{0}^{\prime }+\alpha_{1}^{\prime }G$$

Of interest is the case $\alpha_{1}^{\prime }=\alpha_{1}$, which is known as the proportional odds assumption and has the nice property that dichotomization of *D* gives the logistic regression model for *D* without changing the group effect $\alpha_{1}$, irrespective the cut‐off used for dichotomization.

An alternative ordinal regression model is the adjacent categories model ([29], equation (6.6)). For the present change score this becomes:

(3) $$\mathrm{LN}\left(\frac{P\left(D=1\right)}{P\left(D=0\right)}\right)=\delta_{0}+\delta_{1}G\;\text{and}\;\mathrm{LN}\left(\frac{P\left(D=0\right)}{P\left(D=-1\right)}\right)=\delta_{0}^{\prime }+\delta_{1}^{\prime }G$$

This is in fact the multinomial regression model ([29], equations (6.1) and (6.2)), with *D* = 0 as reference category, and reversed signs of $\delta_{0}^{\prime }$ and $\delta_{1}^{\prime }$ by modeling $\mathrm{LN}\left(\frac{P\left(D=0\right)}{P\left(D=-1\right)}\right)$ instead of $\mathrm{LN}\left(\frac{P\left(D=-1\right)}{P\left(D=0\right)}\right)$. Of interest here is again the case of proportional odds, $\delta_{1}^{\prime }=\delta_{1}$.

As shown in Appendix A, when both models are applied to change scores for binary outcomes, then, defining $\alpha =\left(\alpha_{0}+\alpha_{0}^{\prime }\right)+\left(\alpha_{1}+\alpha_{1}^{\prime }\right)G$ and $\delta =\left(\delta_{0}+\delta_{0}^{\prime }\right)+\left(\delta_{1}+\delta_{1}^{\prime }\right)G$, and writing out $\mathrm{LN}\left(\frac{P\left(D=1\right)}{P\left(D=-1\right)}\right)$ for both models, gives 0<δ<α if $\mathrm{LN}\left(\frac{P\left(D=1\right)}{P\left(D=-1\right)}\right)>0$, and gives 0>δ>α if $\mathrm{LN}\left(\frac{P\left(D=1\right)}{P\left(D=-1\right)}\right)<0$, for *G* = 0 and likewise for *G* = 1. This suggests that the group effect according to model (3) will have the same sign as, but be smaller in absolute value than, the group effect in model (2).

Finally, note that under the proportional odds assumption the adjacent categories model implies that $\left(\frac{P\left(D=1\right)}{P\left(D=-1\right)}\right)=$ $\left(\delta_{0}+\delta_{0}^{\prime }\right)+2\delta_{1}G$, and so, the group effect on change can also be inferred with logistic regression by excluding all cases where *D* = 0, on the understanding that we then estimate $2\delta_{1}$ instead of $\delta_{1}$. This simple method is included into the scenario analyses and expected to give (almost) the same estimate of $\delta_{1}$ as model (3) with the proportional odds assumption if that assumption is valid, but perhaps with a larger standard error. On the one hand, (3) with proportional odds may benefit from pooled estimation by using both equations in (3) and thus also the cases with *D* = 0. On the other hand, cases with *D* = 0 may perhaps not add information for inference on $\delta_{1}$, but only be useful to test the proportional odds assumption. This would then be analogous to the role of the middle value of a trichotomous predictor in polynomial contrast analysis, which does not contribute to inference on the linear contrast, but to testing the linearity assumption. Without the proportional odds assumption, model (3) can be expected to give the same estimate of the group effect on the $LN\left(\frac{P\left(D=1\right)}{P\left(D=-1\right)}\right)$, which is then $\left(\delta_{1}+\delta_{1}^{\prime }\right)$, and the same standard error, as the simple logistic regression excluding the cases *D* = 0. Incidentally, by ignoring the cases *D* = 0, the simple method extends the classical McNemar test for the difference between two paired proportions in a single sample to a test of the difference between two unpaired samples (here the two groups) with respect to the within‐sample difference between two paired proportions (here the two time points). After all, the McNemar test tests whether $\mathrm{LN}\left(\frac{P\left(D=1\right)}{P\left(D=-1\right)}\right)$ = 0, and thus whether $\left(\delta_{0}+\delta_{0}^{\prime }\right)=0$(taking *G* = 0).

### 3.3 Mixed Logistic Regression for Repeated Measures

This method treats the baseline and outcome as repeated measures, denoted as $Y_{i0}$ and $Y_{i1}$ for person *i*, and analyzes these with the following mixed logistic regression model:

(4) $$\mathrm{LN}\left(\frac{P\left(Y_{\mathrm{it}}=1\right)}{P\left(Y_{\mathrm{it}}=0\right)}\right)=\gamma_{0}+\gamma_{1}G+\gamma_{2}T+\gamma_{3}\mathrm{GT}+u_{i}$$

Here, $Y_{\mathrm{it}}$ is the measurement of person *i* at time point *t* (0 for baseline, 1 for outcome), and *G* again is group (1 for treated or exposed, 0 for control), and *T* is time (0 for baseline, 1 for outcome). Finally, $u_{i}$ is a random person effect. The effect of interest is now $\gamma_{3}$, the group by time interaction effect. For continuous outcomes analyzed with mixed linear regression, this effect corresponds to the group difference in change from baseline. For binary outcomes, it is the group difference in change on the logodds scale. Specifically, as shown in Appendix B, the interaction effect $\gamma_{3}$ in Equation (4) corresponds to the group effect 2$\delta_{1}$ on $\mathrm{LN}\left(\frac{P\left(D=1\right)}{P\left(D=-1\right)}\right)$ in the adjacent categories model (3) with proportional odds, and thus also to the group effect in logistic regression of change on group excluding all *D* = 0 cases. This suggests the latter method as a simple alternative to mixed logistic regression for the present pre‐post design. Finally, $\gamma_{2}$ in (4) is the $\mathrm{LN}\left(\frac{P\left(D=1\right)}{P\left(D=-1\right)}\right)$ in the control group (*G* = 0), which is the intercept $\left(\delta_{0}+\delta_{0}^{\prime }\right)$ in the adjacent categories model (3).

Now, for continuous outcomes analyzed with mixed linear regression with an unstructured covariance matrix of the repeated measures, the following has been shown [16, 17, 22]: If group and baseline are independent (i.e., the groups do not differ at baseline, $\gamma_{1}=0$), then the group by interaction effect $\gamma_{3}$ corresponds to the group difference in mean outcome after treatment or exposure, adjusted for the baseline as a covariate. Further, the adjusted and unadjusted group effect on the outcome are then also equal [17], p. 906. These equivalences hold at the population level, as independence of group and baseline rarely holds perfectly at the sample level, even in a RCT. However, for binary outcomes analyzed with logistic regression (or odds ratios), the literature on collapsibility [30, 31, 32] suggests that the adjusted (for the baseline) group effect on the outcome equals the unadjusted group effect on the outcome if group and baseline are independent conditional on the outcome rather than unconditionally as for continuous outcomes. This raises the question whether the group by time effect $\gamma_{3}$ in Equation (4), and thus also the group effect 2$\delta_{1}$ in Equation (3) with proportional odds, equals the group effect $\beta_{1}$ in Equation (1) if group and baseline are independent conditional on the outcome. As shown in Appendix C, this is indeed the case. Further, although conditional independence does not imply marginal independence, it does imply that the marginal dependence between group and baseline is usually weak. This is because, denoting Pearson's correlation as *ρ*, and denoting group, baseline, and outcome as *G*, *X*, and *Y* respectively, we have under conditional independence: $\rho_{\mathrm{GX}.Y}=0$, which implies $\rho_{\mathrm{GX}}=\rho_{\mathrm{GY}}\times \rho_{\mathrm{XY}}$ [33], p. 339. So, under conditional independence of group and baseline, their marginal correlation is the product of two other correlations and thus (much) smaller than each of these two.

### 3.4 Generalized Estimating Equations (GEE) for Repeated Measures

This method differs from the mixed logistic model (4) by leaving the random person effect $u_{i}$ out of the model. Instead, *GEE* accounts for correlation between the repeated measures by assuming some ad‐hoc residual correlation working matrix, such as unstructured or exchangeable. *GEE* thereby gives marginal outcome probabilities as opposed to the subject‐specific outcome probabilities of mixed logistic regression. As shown by various authors (e.g., [34], p. 1054; [35], p. 316), the regression weights in *GEE* are closer to zero than the corresponding weights in mixed logistic regression due to this difference between marginal and subject‐specific (i.e., conditional on the random person effect) probabilities. Specifically, the ratio of the regression weights in the mixed logistic model (4) with random intercept to the weights in the corresponding *GEE* model is approximately $\sqrt{1+\left(\sigma^{2}/2.89\right)}$, where $\sigma^{2}$ is the random intercept variance. The constant 2.89 (= 1.7 × 1.7) stems from the mathematical relation between the logistic and standard normal distribution [36].

## 4 Methods Comparison by Causal Diagrams and Estimands

Before comparing the different methods of analysis on fictitious and real data, their relation to modern concepts of causal inference on treatment effects, specifically estimands, the average causal effect, and causal diagrams, needs to be clarified. Following [37], the estimand can be defined as the effect that a study aims at quantifying for a given treatment on a given outcome in a given (patient) population. In the present paper, effect will be defined as the average causal effect (ACE), following [13, 38]. Let $\Delta_{i}$ be the individual causal effect (ICE) for person *i*, defined as the difference between two potential outcomes *Y*_*i*_ for that person: under treatment (*G* = 1) and under control (*G* = 0), where *G* means group. The ACE is then defined as Δ=E(Y|G=1)−E(Y|G=0), which is the average of all $\Delta_{i}$ in the population. The individual treatment effects $\Delta_{i}$ are unidentifiable then unless strong and (almost) untestable assumptions are made. But, at least in a RCT the ACE is identifiable because, due to the randomized treatment assignment, the average outcome of the controls equals the unobserved (counterfactual) average outcome of the treated had they not been treated, apart from sampling error. Further, in a RCT with a quantitative outcome and absence of intercurrent events such as non‐compliance with treatment and drop‐out the ACE is the same for both methods, regressing the outcome on treatment and baseline, and regressing change from baseline on treatment, although the first method is more efficient [17]. In what follows, complications like non‐compliance and drop‐out will be ignored.

For binary outcomes the ACE definition depends on the method of analysis. For logistic regression of outcome on treatment and baseline, we have: $\Delta =\ln\frac{P\left(Y=1|G=1,X=x\right)}{P\left(Y=0|G=1,X=x\right)}-\ln\frac{P\left(Y=1|G=0,X=x\right)}{P\left(Y=0|G=0,\;X=x\right)}$, which is $\beta_{1}$ in model (1) if there is no confounding, as in a RCT. This ACE applies to *x* = 0 as well as *x* = 1, because (1) assumes absence of interaction between treatment *G* and baseline *X*. For ordinal regression of change from baseline with the adjacent categories with proportional odds model (3), we have: $\Delta =\ln\frac{P\left(D=1|G=1\right)}{P\left(D=-1|G=1\right)}-\ln\frac{P\left(D=1|G=0\right)}{P\left(D=-1|G=0\right)}$, which is $2\delta_{1}$ if there is no confounding. Now, as shown in Section 3, $\beta_{1}=2\delta_{1}$ if and only if treatment and baseline are independent conditional on the outcome. In a RCT, however, we have marginal independence instead. So, (1) and (3) target somewhat different estimands.

**FIGURE 2:**
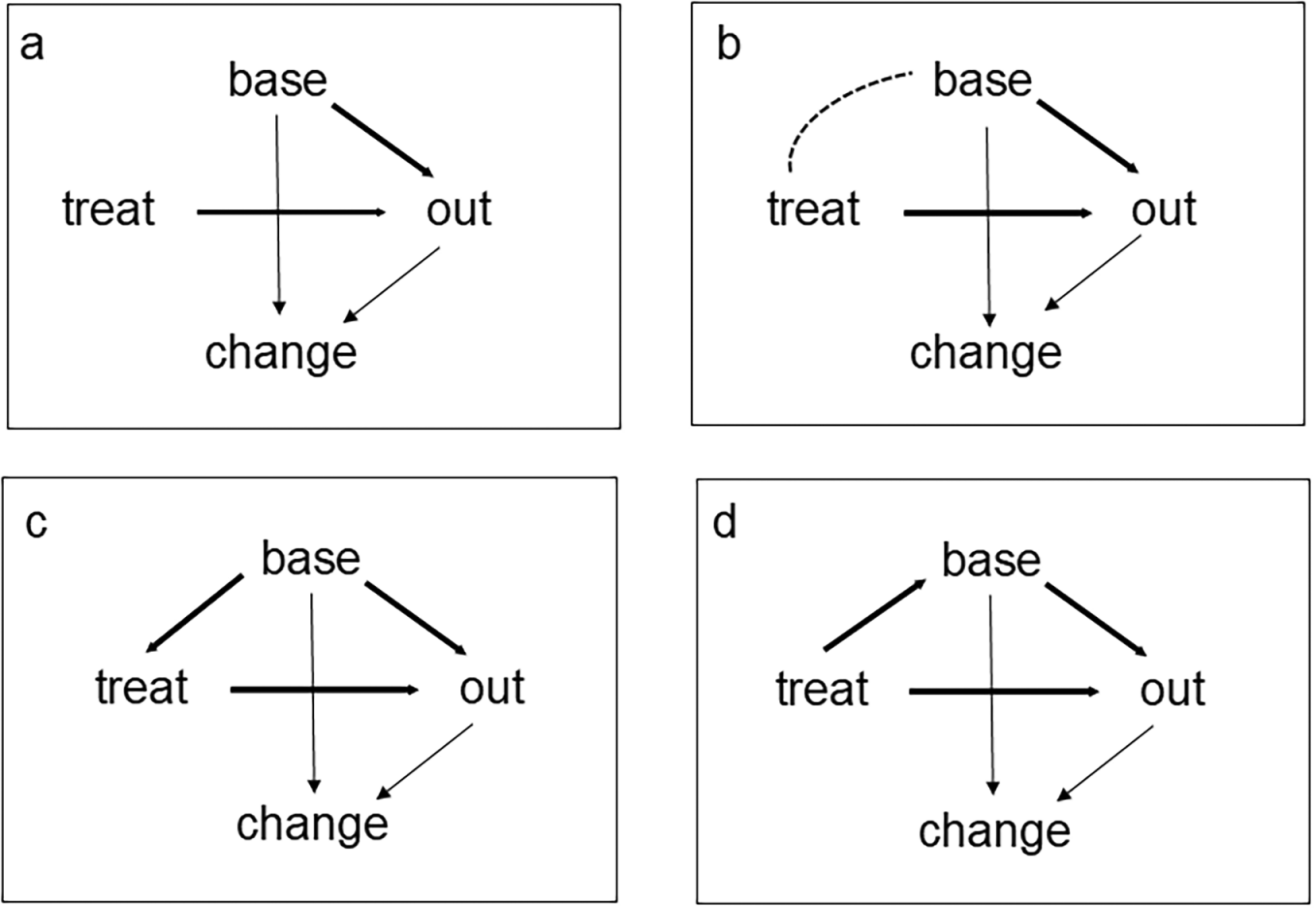
Causal diagrams of relations between treatment, baseline, and outcome.

For other study designs than the RCT unbiased estimation of the ACE depends on a correct identification of the causal relations between treatment, baseline, outcome, and other variables deemed relevant, and on valid and reliable measurement of those other variables that need to be adjusted for in the analysis. An important tool for that are causal diagrams, also known as directed acyclic graphs (DAGS) [9, 28]. These have been known for at least 50 years in the social sciences under the headings path analysis and structural equation modeling [39], and have more recently gained popularity and importance in biostatistics and epidemiology as tools to make the causality assumptions of a researcher explicit and thereby to find out which variables need to be adjusted for and which not [9, 28]. Ignoring at first all other variables and focusing on treatment, baseline, and outcome, four plausible causal diagrams are shown in Figure 2. Panels (c) and (d) are discussed in [9], extending both with paths from baseline and outcome to an additional variable, change from baseline (or “gain” as in [9]). These extensions are included into the panels as thin lines. Whether the relations between baseline, outcome, and change are best modeled as causal paths or as mutual dependencies on a common cause of which the measures are fallible proxies is debatable. For now, the approach of [9] is followed, but we get back to this issue at the end of this Section.

Panel (a) in Figure 2 applies to RCTs. Due to randomized assignment there is neither a causal relation nor even a correlation between treatment and baseline, at least provided the baseline measurement is done before treatment assignment. As said before, in a RCT we have marginal independence between group and outcome. However, as shown in Section 3, the estimands of baseline adjusted regression of outcome on group with Equation (1), and ordinal regression of change on group with Equation (3) and proportional odds, are the same only under conditional independence of group and baseline given the outcome. So, in a RCT the estimands of (1) and (3) are not the same, that is, $\beta_{1}\neq$ $2\delta_{1}$. Further, except under conditional independence of group and baseline given the outcome, both estimands also differ from that of unadjusted logistic regression of the outcome on group ignoring the baseline. In particular, under marginal independence of group and baseline, as in panel (a), the unadjusted group effect on the outcome is a bit closer to zero than the baseline adjusted group effect on the outcome [23, 32]. This is different from the case of a quantitative outcome analyzed with linear regression, where the estimand is the same for all three methods (unadjusted, adjusted, change) when applied to a RCT. But, at least, all three methods for a binary outcome unbiasedly estimate their own estimand in a RCT.

Panel (b) in Figure 2 assumes some correlation, but no direct causal link, between group and baseline. Such correlation can arise in a nonrandomized study when treatment assignment and baseline share a common cause. As an example, if study participants are allowed to choose their own treatment and their choice as well as their baseline depends on their income, then a correlation between treatment and baseline arises. If, conditional on income, treatment assignment is at random, then adjustment for income rather than for baseline is needed for unbiased estimation of the treatment effect on the outcome. The differences between outcome analysis ignoring the baseline, and outcome analysis with the baseline as covariate, and change from baseline, would then be as in a RCT as long as we adjust for income in all three methods. So, the three methods would all give unbiased estimation, but of different estimands. Whether treatment assignment is really at random, given income, is of course another matter. At best, propensity scoring methods can test whether any other measured variable obtained before treatment assignment is related to that assignment and if so, we also need to adjust for that variable [40, 41, 42]. But, there is always a risk of unmeasured confounders. Further, with treatment assignment of natural groups, such as community A being treated and community B serving as control in a health promotion campaign, the confounder community is collinear with treatment and cannot be adjusted for.

Panel (c) in Figure 2 assumes that the baseline affects treatment assignment, as is the case if each patient's treatment is chosen (by patient or clinician) based on the patient's baseline value. For instance, in choosing between medication and psychotherapy for treating depression, a patient may be more inclined to choose medication as the baseline depression is more severe. An extreme case of this is the regression discontinuity design (RDD, [12]) or cut‐off design [15], where all patients with baseline value above a certain cut‐off are treated and all patients below the cut‐off are controls, or vice versa. Such a deterministic design might satisfy the need felt by clinicians and patients to first serve those most in need of treatment. But, it is already tricky for quantitative outcomes because of its strong assumptions of linearity and additivity of treatment and baseline effects that can hardly be tested with sufficient power due to the strong correlation between treatment and baseline. For binary outcomes, unbiased effect estimation with a RDD is even impossible due to collinearity between treatment and baseline which prevents adjustment. However, the weaker version where treatment assignment depends in a probabilistic way on baseline is feasible and will be included into the scenarios in the next section. Provided treatment assignment is at random conditional on the baseline, as assumed in panel (c), covariate adjustment following model (2) ensures unbiased estimation of the ACE, whereas change analysis suffers from regression to the mean as already known for quantitative outcomes [4, 14, 15, 17]. In terms of panel (c), the bias in change analysis arises because it does not correct for the baseline, which affects treatment assignment as well as the change score and thus is a confounder. In fact, in ordinal regression of change on treatment group with model (2) or (3) adding the baseline as covariate would give an unidentifiable model, both with and without proportional odds assumption. This is because *D* = −1 cannot occur if baseline *X* = 0, and *D* = +1 cannot occur if baseline *X* = 1, so that only the first equation in (2) respectively (3) applies for *X* = 0 and only the second equation applies for *X* = 1, and the two equations for a given model have different intercepts already. Adding *X* as covariate would make its regression weight inseparable from the intercept in the second equation.

Finally, panel (d) lets treatment assignment affect the baseline. This diagram was used in [9] for the case where group was not treatment but biological sex, one of the examples in the original publications on Lord's paradox [1, 2], and it made sense there. In the present paper, where group is a treatment, panel (d) can only apply if the baseline measurement takes place after treatment assignment, a procedure that is not to be recommended for two reasons that are most easily understood in case of a RCT. First, if the baseline measurement precedes treatment assignment and patients drop out after treatment assignment but before outcome measurement, they can be included into an intention to treat analysis with mixed regression for repeated measures using their baseline values to prevent bias arising from baseline‐dependent dropout (a case of missingness at random). This is not possible with baseline measurement after treatment assignment. Second, if the baseline is measured before treatment assignment and the two are unrelated as in a RCT, then adjusting for the baseline increases power and precision of treatment effect estimation. But, if the baseline is affected by treatment (assignment) as in panel (d), then adjusting for the baseline changes the estimand from the total treatment effect on the outcome into the so‐called direct effect, which is the total effect minus the effect mediated by the baseline measurement. For instance, the treatment (assignment) may affect the patient's confidence in recovery and thereby baseline health, which in turn may affect the health outcome, especially if the outcome of interest is a self‐report. The question then is which estimand is of interest, the total effect or the direct effect. Moreover, the total effect can be estimated unbiasedly in a RCT, at least assuming absence of non‐compliance and of missingness not at random (MNAR). But, direct effect estimation by adjusting for a baseline measured after treatment assignment can introduce bias arising from an unmeasured confounder that affects both baseline and outcome and thereby the mediated effect estimation. Examples of such confounders are life style and genetic makeup. Relatedly, measurement error in the sense of fluctuations in the patient's state attenuates the correlation between baseline and outcome depending on the time interval between both measurements. This leads to underestimation of the mediated effect and overestimation of the direct effect. In short, baseline measurement after treatment assignment is to be avoided, and panel (d) will not be further discussed in this paper.

All panels in Figure 2 can be extended with observed and unobserved extra variables, leading to a multitude of causal diagrams and recommendations for when to adjust and when not [28]. Relatedly, one may ask whether the relations between baseline, outcome, and change should be represented as causal paths or as correlations instead, arising from their common dependence on a latent variable that may change over time, as done in structural equations modeling. However, these extensions of Figure 2 are beyond the scope of this paper, which focuses on the relations between the various methods in Section 3 and on the occurrence of Lord's paradox for binary outcomes. As a final note, in studies where patients choose their own treatment it may be difficult to tell whether panel (b) or (c) applies.

## 5 Methods Comparison on Fictitious Data

### 5.1 Five Fictitious Data Scenarios

Five data sets were constructed, each with a sample size of *N* = 1000 per group to minimize the role of sampling error, differing from each other in the presence of a baseline group difference, and of a group difference in change over time on the logodds scale. The purpose was two‐fold. First, to demonstrate Lord's paradox for a binary outcome. Secondly, to illustrate the mathematical relations in Appendices B and C, that is, (a) the equivalence between the group effect 2$\delta_{1}$ in model (3) with proportional odds and the group by time effect $\gamma_{3}$ in model (4) for all scenarios, and (b) the equivalence between the group effect $\beta_{1}$ in model (1) and $\gamma_{3}$ in model (4) in case of conditional independence of group and baseline given the outcome, and relatedly, the similarity between $\beta_{1}$ and $\gamma_{3}$ under marginal independence of group and baseline ($\gamma_{1}=0$). Specifically, the following five scenarios were constructed:

1. Natural groups with a baseline difference, and parallel change on the logodds scale.
2. Natural groups with a baseline difference, and convergence on the logodds scale.
3. A RCT: No group difference at baseline (i.e., marginal independence between group and baseline), but a group difference in change on the logodds scale (divergence).
4. A probabilistic version of the regression discontinuity design (RDD): persons are assigned to a group based on their baseline value (70% of those with baseline value 1 are assigned to treatment, against only 30% of those with baseline value 0). In the total sample of 2000 persons, there is no change from baseline to outcome.
5. Conditional independence between group and baseline given the outcome.

**FIGURE 3:**
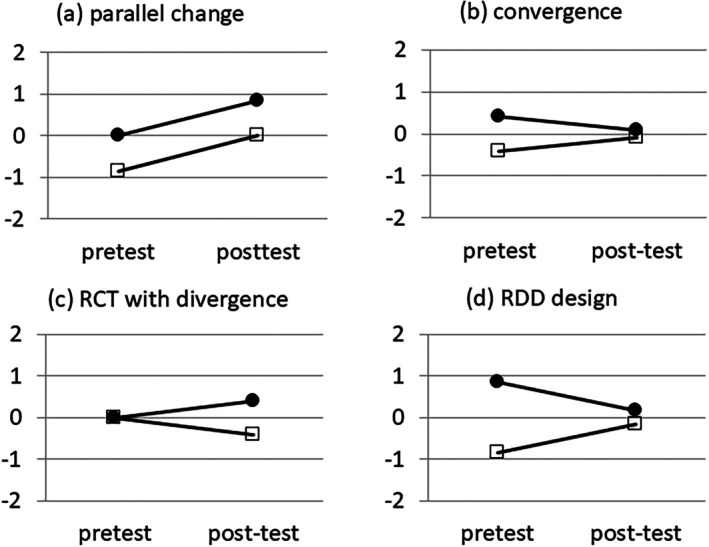
Logodds (vertical axis) against time (horizontal axis) per group (⚫ = treated, □ = control) in four scenarios.

The first four scenarios are shown in Figure 3, the fifth scenario resembles the third one, and the data for all five scenarios are found in Appendix D. The data were such that the methods could be compared in terms of a single parameter per method, that is, (a) in terms of covariate adjustment, there was no group by baseline interaction, and (b) in terms of ordinal regression of change, proportional odds were satisfied. Further, the within‐group association between baseline and outcome was similar in all scenarios, with a correlation between 0.17 and 0.20, and an odds ratio between 2.00 and 2.67. The reason for choosing the first four scenarios was as follows: based on published work on continuous outcomes [16, 17]: In a RCT both covariate adjustment and change from baseline are valid methods, but the first has more power. For the RDD, only covariate adjustment is valid, whereas change from baseline ignores the regression to the mean effect (for this effect for binary data, see [43]). For the two natural groups scenarios it is not clear which method is valid when. Ignoring the role of sampling error by taking a large sample, Lord's paradox can occur in both scenarios. The fifth scenario served to illustrate the equivalence between mixed logistic regression and covariate adjustment under conditional independence of group and baseline. Note that, in terms of the causal diagrams in Figure 2, scenarios (1) and (2) fit panel (b), scenario (3) fits panel (a), and scenario (4) fits panel (c). As said before, in studies where patients choose their own treatment, it may be difficult to tell whether panel (b) or (c) applies.

Three further data sets were generated (see Appendix E) to explore (a) the effect of a stronger baseline‐outcome correlation on the differences between covariate adjustment and change analysis and (b) the effect of violation of the proportional odds assumption.

### 5.2 Results

Each data set was analyzed with the methods discussed before, using SPSS version 28 and R version 4.4.1. Specifically, logistic regression of the outcome on group and baseline, logistic regression of change on group excluding ‘ties’ (i.e., cases with zero change, *D* = 0), ordinal regression of change on group with the cumulative logit model, and *GEE* were applied with SPSS. Ordinal regression of change on group with the adjacent categories model with proportional odds, which is not available in SPSS, was run with R, using the vglm() function in the vgam package. Mixed logistic regression, which is biased in SPSS due to using PQL1 estimation [44, 45], was run with R, using the glmer() function with adaptive Gauss‐Hermite quadrature and seven quadrature points in the lme4 package.

The results of the four scenarios in Figure 3 and of the fifth scenario (conditional independence of group and baseline) are summarized in Table 1, with scenarios as columns and methods as rows. Comparing between methods per scenario shows the following things:

**TABLE 1:** Analysis of five fictitious group comparisons with six methods of analysis (*B* = estimated group effect for classical methods, estimated group by time interaction effect for repeated measures methods).

	Natural groups, parallel change	Natural groups, convergence	RCT, divergence	RDD, no effect	Conditional independence of group and pretest given posttest
Classical methods					
Covariate adjustment	*B* = 0.693	*B* = 0.000	*B* = 0.847	*B* = 0.000	*B* = 1.386
	SE = 0.097	SE = 0.093	SE = 0.094	SE = 0.100	SE = 0.095
	*p* < 0.001	*p* = 1.000	*P* < 0.001	*P* = 1.000	*P* < 0.001
Change (logistic regression excl. zero change)	*B* = 0.000	*B* = −0.811	*B* = 1.022	*B* = −1.694	*B* = 1.386
	SE = 0.163	SE = 0.144	SE = 0.146	SE = 0.154	SE = 0.150
	*p* = 1.000	*p* < 0.001	*P* < 0.001	*P* < 0.001	*P* < 0.001
Change (adjacent categories^a^)	*B* = 0.000	*B* = −0.811	*B* = 1.022	*B* = −1.694	*B* = 1.386
	SE = 0.150	SE = 0.144	SE = 0.146	SE = 0.154	SE = 0.144
	*P* = 1.000	*p* < 0.001	*p* < 0.001	*P* < 0.001	*P* < 0.001
Change (cumulative logits)	*B* = 0.000	*B* = −0.506	*B* = 0.636	*B* = −1.048	*B* = 0.857
	SE = 0.089	SE = 0.090	SE = 0.090	SE = 0.094	SE = 0.088
	*P* = 1.000	*p* < 0.001	*P* < 0.001	*P* < 0.001	*P* < 0.001
Methods for repeated measures					
Mixed logistic^b^ model: time, group × time	*B* = 0.855	*B* = 0.000	*B* = 1.022	*B* = 0.000	*B* = 1.613
	SE = 0.118	SE = 0.112	SE = 0.116	SE = 0.120	SE = 0.115
Mixed logistic^b^, model: group, time, group × time	*B* = 0.000	*B* = −0.811	*B* = 1.022	*B* = −1.695	*B* = 1.389
	SE = 0.151	SE = 0.144	SE = 0.146	SE = 0.154	SE = 0.145
GEE model: time, group × time	*B* = 0.657	*B* = 0.000	*B* = 0.811	*B* = 0.000	*B* = 1.349
	SE = 0.090	SE = 0.088	SE = 0.089	SE = 0.088	SE = 0.092
GEE model: group, time, group × time	*B* = 0.000	*B* = −0.651	*B* = 0.811	*B* = −1.374	*B* = 1.163
	SE = 0.117	SE = 0.115	SE = 0.114	SE = 0.120	SE = 0.118

^a^ R package vgam, function vglm(), and assuming proportional odds. Both *B* and SE have been multiplied with 2 to obtain the group effect on ln{P(D = 1)/P(D = −1)}.

^b^ R package lme4, function glmer() with adaptive GH quadrature and seven quadrature points. Reported is the estimated group by time effect.

First, covariate adjustment and change from baseline analysis contradict each other with respect to the presence of a group difference on the outcome in all three scenarios where the groups differ at baseline (i.e., scenarios 1, 2, and 4), with one method giving a highly significant group difference, and the other method not even giving a trace of a group effect. Thus, Lord's paradox occurs not only for quantitative outcomes, but likewise for binary ones.

Second, in each scenario the group by time interaction effect estimate ${\hat{\gamma }}_{3}$ in mixed logistic regression with the full model (4) that allows for a baseline group difference (i.e., with $\gamma_{1}$ unconstrained) is equal to the group effect estimate $2{\hat{\delta }}_{1}$ in ordinal regression of change using the adjacent categories model (3) with proportional odds, and is also equal to the group effect estimate in logistic regression of change excluding cases with zero change. These three methods also give the same standard errors, apart from a slightly larger SE for the logistic regression of change in scenarios 1 and 5. Thus, it seems like the equivalences between parameters shown in Section 3 and Appendix B, also hold for their estimates and SEs, although a simulation study would be needed to check this.

Third, the group by time interaction effect estimate ${\hat{\gamma }}_{3}$ in mixed logistic regression with a constrained model that assumes absence of a baseline group difference (i.e., with $\gamma_{1}$ = 0, marginal independence of group and baseline) is similar to, but larger than, the group effect ${\hat{\beta }}_{1}$ in logistic regression of outcome on group and baseline (i.e., covariate adjustment) in terms of effect estimate and standard error. The equivalence between mixed linear regression and ANCOVA shown for continuous outcomes in [17, 22] thus does not hold strictly for the binary case. Instead, as shown in Section 3 and Appendix C, and illustrated by scenario 5, $\gamma_{3}$ in the full mixed model (4) without constraint on $\gamma_{1}$ equals $\beta_{1}$ in the covariate adjustment model (1) under conditional independence of group and baseline given the outcome.

Fourth and related to the previous point, the group by time effect in *GEE* with the constraint $\gamma_{1}$ = 0 is almost the same as the group effect in the logistic regression of the outcome on group and baseline in all scenarios. The published equivalence between ANCOVA and the constrained ($\gamma_{1}$ = 0) mixed linear regression for continuous outcomes with unstructured covariance matrix of the repeated measures (e.g., assuming a random intercept and allowing baseline and outcome to have different variances) is not at variance with this, given that this mixed linear model implies the same mean and variance structure as a *GEE* model with identity link and unstructured covariance matrix. Concerning the relation between the present mixed logistic regression and *GEE* results it may furthermore be added that the observed ratio of their respective group by time effect estimates in Table 1 is close to the theoretically expected $\sqrt{1+\left(\sigma^{2}/2.89\right)}$, where $\sigma^{2}$ is the random intercept variance, in all scenarios, as ${\hat{\sigma }}^{2}$ varied from 1.56 in scenario 1 to 0.96 in scenario 5, with values from 1.2 to 1.3 for the other three scenarios. In contrast, mixed logistic regression with SPSS (not shown here) gave effect estimates and standard errors that differed less than 10% from those of *GEE*, thus confirming the known downward bias of PQL1 estimation, which is the method used by SPSS for generalized linear mixed models.

Fifth and last, comparing the results of ordinal regression of change for the cumulative logit model (2) with those for the adjacent categories model (3) shows that (2) gives a group effect that is smaller than, but larger than half, the group effect according to (3) in all scenarios. This is in line with Section 3 and Appendix A, noting that Table 1 reports $2{\hat{\delta }}_{1}$ instead of ${\hat{\delta }}_{1}$ for the adjacent categories model (3) for the sake of comparison with ${\hat{\gamma }}_{3}$ from model (4).

As said in Section 5.1, three further data sets, given in Appendix E, were analyzed with the same methods, and the results of these can be summarized as follows:

The first two extra data sets were similar to the first two in Appendix D, respectively the parallel change and the convergence scenarios, except that the baseline‐outcome correlation within groups was stronger now, with a correlation of 0.41 (odds ratio of 6) respectively 0.49 (odds ratio of 9) instead of 0.20. The reason behind this was the fact that, for quantitative outcomes, the discrepancy between covariate adjustment (ANCOVA) and change analysis of groups differing at baseline decreases as this correlation increases because the regression weight of the baseline as covariate then moves toward one. The results of the extra data sets were only partly in line with this, as Lord's paradox still occurred, albeit less strongly than in Table 1 for the parallel change scenario (i.e., the effect of group according to covariate adjustment was now only half as large as in Table 1). The role of the baseline‐outcome correlation in Lord's paradox for binary data thus needs further study.

The third extra data set was a modification of the first one in Appendix D such that the proportional odds assumption for adjacent categories regression of change was violated. Applying adjacent categories regression with the, now invalid, proportional odds assumption gave a group effect on $\mathrm{LN}\left(\frac{P\left(D=1\right)}{P\left(D=-1\right)}\;\right)$ that differed from that of logistic regression of change excluding cases with zero change (respectively *B* = −0.291, SE = 0.150 vs. *B* = 0.000, SE = 0.163, to compare: covariate adjustment gave: *B* = 1.386, SE = 0.105). Adjacent categories regression without the proportional odds assumption (which is multinomial regression) gave the same group effect and SE for $\mathrm{LN}\left(\frac{P\left(D=1\right)}{P\left(D=-1\right)}\;\right)$ as logistic regression of change excluding cases with zero change in this and all other data sets.

As a final remark, one might perhaps consider combining methods into “the best of both worlds” by adding the baseline as covariate to the ordinal regression of change from baseline on group. For quantitative outcomes analyzed with ANCOVA, this gives the same group effect estimate and standard error as regressing the outcome instead of change on group and baseline. This follows trivially from the ANCOVA equation for regressing change on group and baseline and then adding the baseline on both sides of the equation. For binary outcomes analyzed with either of the two ordinal regression models (2) and (3) for change, adding the baseline as covariate gives an unidentifiable model, as explained in Section 4 when discussing panel (c) of Figure 2.

## 6 Results of the Smoking Prevention Study

With the results in Sections 3 and 5 in mind, let us return to the smoking prevention study [26], see Figure 1. Using the same methods as in Sections 3 and 5, this section compares the out‐of‐school tailored intervention with the control condition with respect to current smoking at baseline (T0), 5 months later (T1), and again 4 months later (T2). The four‐way contingency table of current smoking per time point and treatment arm (out‐of‐school, control) is provided in Appendix F. The results of the statistical analyses are given in Table 2, ignoring at first the clustering of pupils within schools, to which we get back later on. Not shown in Table 2 but reported here for completeness are the comparisons between intervention and control per time point. Ignoring the clustering, the difference in smoking percentage was significant at T0 (*p* = 0.016) and T1 (*p* = 0.005), but not at T2 (*p* = 0.927). Accounting for the clustering by using *GEE* with an exchangeable covariance structure gave *p* = 0.028, 0.027, and 0.926, respectively, thus confirming that the two treatment arms differed at T0 and T1, but not at T2, in line with Figure 1.

**TABLE 2:** Analysis of the smoking prevention study with five methods of analysis (*B* = estimated group effect for classical methods, estimated group by time interaction effect for repeated measures methods). Analysis per pair of time points (T0 = baseline, T1 = outcome, T2 = follow‐up).

	T0 – T1 (parallel change)	T0—T2 (convergence)	T1‐T2 (convergence)
Classical methods			
Covariate adjustment	*B* = −0.431	*B* = 0.140	*B* = 0.274
	SE = 0.200	SE = 0.192	SE = 0.216
	*p* = 0.031	*p* = 0.468	*p* = 0.204
Change (logistic regression excl. zero change)	*B* = 0.004	*B* = 0.748	*B* = 0.967
	SE = 0.349	SE = 0.334	SE = 0.378
	*p* = 0.990	*p* = 0.025	*p* = 0.010
Change (adjacent categories^a^)	*B* = −0.175	*B* = 0.663	*B* = 0.938
	SE = 0.336	SE = 0.327	SE = 0.376
	*p* = 0.601	*p* = 0.043	*p* = 0.013
Methods for repeated measures			
Mixed logistic^b^	*B* = −0.593	*B* = 0.221	*B* = 0.421
Model:	SE = 0.279	SE = 0.265	SE = 0.309
Time, group × time	*p* = 0.033	*p* = 0.404	*p* = 0.174
Mixed logistic,^b^	*B* = −0.097	*B* = 0.696	*B* = 0.965
model:	SE = 0.342	SE = 0.330	SE = 0.371
Group, time, group × time	*p* = 0.776	*p* = 0.035	*P* = 0.009
GEE	*B* = −0.385	*B* = 0.125	*B* = 0.211
Model:	SE = 0.176	SE = 0.175	SE = 0.167
Time, group × time	*p* = 0.029	*p* = 0.476	*p* = 0.203
GEE	*B* = −0.056	*B* = 0.471	*B* = 0.519
Model:	SE = 0.220	SE = 0.225	SE = 0.197
Group, time, group × time	*p* = 0.801	*p* = 0.036	*p* = 0.008

^a^ R package vgam, function vglm(), and assuming proportional odds. Both B and SE have been multiplied with 2 to obtain the group effect on ln{P(D = 1)/P(D = −1)}.

^b^ R package lme4, function glmer() with adaptive GH quadrature and seven quadrature points. Reported is the estimated group by time effect.

Focusing now on the method comparisons in Table 2, the following can be seen:

First, covariate adjustment and change from baseline analysis contradict each other with respect to the group difference on the outcome in each pair of time points, with covariate adjustment showing a difference for the time pair T0‐T1 and change showing a difference for both other time pairs. This is in line with the results in Section 5.2 and with Figure 1, where the two treatment arms change in parallel from T0 to T1, and converge at T2. A difference with Section 5.2 is that change analysis excluding zeros and ordinal regression no longer give the same results, although they are still similar. This is due to a violation of the proportional odds assumption in the smoking data for all time pairs, which was highly significant for the time pair (T0, T1). Repeating the ordinal regression without that assumption gave the same group effect on $\mathrm{LN}\left(\frac{P\left(D=1\right)}{P\left(D=-1\right)}\;\right)$ as change analysis excluding zeros for every time pair.

Second, for each time pair the group by time interaction effect ${\hat{\gamma }}_{3}$ in mixed logistic regression with the full model (4) is close to the group effect $2{\hat{\delta }}_{1}$ in ordinal regression of change using the adjacent categories model (3) with proportional odds, and also to the group effect in logistic regression of change excluding cases with zero change. That mixed logistic regression differs a bit from both other methods may partly be due to the difference in handling missing data, with mixed logistic regression using all available data and both other methods using including only complete cases. Appendix F shows about 10% missingness, depending on group and time point. Additionally, mixed regression may differ from ordinal regression of change due to violation of the proportional odds assumption, and from analysis of change excluding zeroes due to the high percentage of zero changes, about 90%, see Appendix F.

Third, the group by time effect ${\hat{\gamma }}_{3}$ in the constrained mixed logistic model (i.e., with $\gamma_{1}$ = 0) is similar to the group effect ${\hat{\beta }}_{1}$ in logistic regression of outcome on group and baseline (i.e., covariate adjustment) in terms of sign and significance yes/no. Their differences in effect estimate and standard error may be due to the difference in handling missingness and, just like in Section 5.2, also to the difference between marginal independence of group and covariate (i.e., $\gamma_{1}$ = 0) and their independence conditional on the outcome. For details, see Appendix C and Section 3.

Fourth and last, the *GEE* results are similar to those of mixed logistic regression, differing in effect size in line with the known relation between the two methods [34], p. 1054; [35], p. 316.

The above results serve to illustrate the occurrence of Lord's paradox in real data, but to do justice to these specific data, the clustering of pupils within schools must be accounted for, which was ignored in Table 2. Table 3 (first two rows) therefore shows the results of the first two methods, covariate adjustment and analysis of change excluding zero changes, when obtained with *GEE* with pupils clustered in schools with an exchangeable covariance structure. The effect estimates are similar to those in Table 2, but the standard errors are larger due to the design effect arising from the clustering. As a result, the significant group difference at T0‐T1 according to covariate adjustment and at T0‐T2 according to change are no longer significant, although the latter is still close to significance. Further, the group difference at T1‐T2 according to change is still significant, thus confirming Lord's paradox for that pair of time points. Note that the first two pairs are most relevant to cluster randomized trials by using T0 as baseline, while the last pair is relevant for observational studies by using T1 as baseline, and this pair furthermore fits panel (d) in Figure 2.

**TABLE 3:** Analysis per pair of time points of measurement in the smoking prevention study, accounting for clustering of pupils in schools with exchangeable covariance matrix (classical methods only).

	T0 – T1 (parallel change)	T0—T2 (convergence)	T1‐T2 (convergence)
Covariate adjustment with clustering (GEE)	*B* = −0.408	*B* = 0.134	*B* = 0.284
	SE = 0.263	SE = 0.247	SE = 0.261
	*p* = 0.121	*p* = 0.587	*p* = 0.276
Change (logistic regression excl. zero change) with clustering (GEE)	*B* = −0.054	*B* = 0.666	*B* = 1.032
	SE = 0.406	SE = 0.366	SE = 0.448
	*p* = 0.895	*p* = 0.069	*p* = 0.021
Covariate adjustment with clustering (GEE) and contextual effect	*B* = −0.289	*B* = 0.328	*B* = 0.389
	SE = 0.267	SE = 0.251	SE = 0.267
	*p* = 0.278	*p* = 0.191	*p* = 0.145

Given the clustered data structure, the covariate adjustment method can be extended with a so‐called contextual effect by adding the cluster mean baseline as a covariate to the model to allow for a difference between within‐cluster and between‐cluster regression of outcome on baseline ([46] p. 12, [47] p. 29). A significant contextual effect was found for the time pair T0–T2 (*p* = 0.009), and a trend for the other two time pairs (*p* < 0.10). The results of this analysis for the treatment effect are shown in the last row of Table 3 and are in line with the covariate adjustment method without contextual effect, differing from it in effect estimate and *p*‐value but not in conclusion.

## 7 Discussion

It has long been known that covariate adjustment and change analysis can give contradictory results when applied to a nonrandomized group comparison with a baseline difference on a quantitative outcome. It has also been shown in the literature on quantitative outcomes that covariate adjustment gives the same results as a model treating baseline and outcome as repeated measures and assuming absence of a group difference at baseline. The present paper extends those results to the case of a binary outcome, such as smoking or the presence of a specific health problem, by comparing classical methods (logistic and ordinal regression) and modern methods (mixed logistic regression, *GEE*), first mathematically, then in terms of causal diagrams, and finally on several constructed scenarios with a large sample. The results are in line with those for quantitative outcomes. Mathematically, the group effect in logistic regression of outcome on group and baseline corresponds to the group by time interaction in mixed logistic regression of both repeated measures if group and baseline are independent conditional on the outcome, and conditional independence implies weak marginal dependence (Appendix C). Also mathematically, the group effect in ordinal regression of change on group with the adjacent categories model with proportional odds assumption corresponds to the group by time interaction in mixed logistic regression, irrespective the relation between group and baseline (Appendix B). The results of the scenarios are roughly in line with these theoretical results. Covariate adjustment (logistic regression of the outcome on group and baseline) gave similar results as mixed logistic regression (4) with the constraint $\gamma_{1}$ = 0 (i.e., assuming absence of a baseline group difference), and almost the same results as *GEE* with the same constraint, in all scenarios. In case of conditional independence of group and baseline given the outcome, covariate adjustment gave the same results as unconstrained mixed logistic regression according to theory (Appendix C) as well as scenario 5. Further, ordinal regression of change on group gave results that were almost equal (adjacent categories model), or else similar (cumulative logit model), to those of unconstrained mixed logistic regression or *GEE* with a model that allows for a baseline group difference. Interestingly, in all scenarios a simple logistic regression of change on group excluding all cases with zero change gave almost the same effect estimate and standard error, as adjacent categories ordinal regression and as unconstrained mixed logistic regression and *GEE*. If this result is confirmed in a future simulation study, it suggests a very simple method for group comparisons on a change in a binary outcome measured twice per person. Finally, analyses of the smoking prevention study gave results similar to those in the scenarios, with the time pair T0‐T1 behaving like scenario 1 (parallel change) and both other time pairs behaving like scenario 2 (convergence).

At first glance, the present mathematical and scenario results might suggest that covariate adjustment is incorrect, and change analysis is correct for the comparison of groups that differ at baseline. Things are more complicated than that, however. First, as the fourth scenario (RDD) illustrates, if groups are constructed after the baseline measurement, and based on that measurement, then covariate adjustment, not change analysis, is the correct method, whether the groups are constructed to differ or not, as already known for quantitative outcomes [15, 16, 17]. In terms of Figure 2, causal diagram (c) then applies. In practice, it may be hard to tell whether diagram c or b applies when patients choose their own treatment. Secondly, change analysis makes a strong assumption, that of equal change on the logodds scale if neither group is treated or exposed. If groups differ at baseline, this suggests that they have changed differently in the past, which raises doubts about the assumption underlying change analysis, especially if the time interval between baseline and outcome is long. Third and last, neither method is guaranteed to be correct for group comparisons other than in randomized trials or designs with group assignment based on the baseline measurement. As in any observational study, careful measurement and adjustment for confounders is required, supported by the use of causal diagrams. If covariate adjustment and change analysis give contradictory results, more sophisticated analysis is needed, or a replication study with multiple control groups or multiple baselines to test the assumption of equal change in the absence of treatment or exposure. If covariate adjustment and change analysis do not contradict each other, this gives some reassurance, but it is no proof of correctness of either method. Even better than using advanced statistical methods would be to prevent the paradox from occurring at all, which requires a (cluster) randomized trial. Obviously, this is an option for the study of treatment effects thought to be beneficial, but not for the study of effects of exposure to substances thought to be harmful.

As is known from the statistical literature on quantitative outcomes, the extent to which covariate adjustment and change from baseline contradict each other in nonrandomized group comparisons depends on the within‐group correlation between baseline and outcome. The weaker this correlation, the stronger the contradiction. Now, this correlation is known to be attenuated by measurement error (continuous outcomes) respectively non‐differential misclassification (binary outcomes). One might therefore consider solving the paradox by trying to correct the correlation and thereby also the regression weight of the baseline covariate for this attenuation effect. However, as shown for quantitative outcomes in [17, 48], such a correction itself depends on an assumption about the true (i.e., without measurement error) group difference at baseline, and depending on the assumption, the correction may lead back to uncorrected covariate adjustment or to change from baseline analysis. Further, as shown by two additional scenarios briefly mentioned in Section 5.2, the contradiction between the two uncorrected methods can still occur in the presence of a strong baseline‐outcome correlation.

This study is limited in several respects. First, it focused on nonrandomized group comparisons because the merit of covariate adjustment in randomized trials with a binary outcome has already been studied [24], and Lord's paradox will not occur in RCTs except due to sampling error or due to a rather unknown small effect of covariate adjustment that occurs in generalized linear models such as logistic regression, but not in linear models [23, 32, 49]. Secondly, in terms of covariate adjustment by logistic regression of the outcome on group and baseline, group by baseline interaction was avoided in all scenarios to obtain a single group effect that could then be compared with the effect obtained with change from baseline. Third, the scenarios were limited to two repeated measurements without missing data. With more than two measurements and/or substantial missingness, the classical methods (logistic and ordinal regression) are not an option anymore and mixed logistic regression or *GEE* is needed. However, that does not solve the issue of how to interpret group by time interaction in the presence of a baseline group difference. Fourth, the present scenarios were small‐scale and only served to (a) demonstrate Lord's paradox for binary data and (b) illustrate the mathematical results in this paper about the relations between the various methods. This leaves some questions unanswered, for instance, why the group effect in covariate adjustment (logistic regression of outcome on group and baseline) was more similar to constrained *GEE* than to constrained mixed logistic regression in all scenarios except conditional independence. Or, why logistic regression of change excluding cases with zero change gave not only the same group effect estimate but also (almost) the same standard error as ordinal regression with model (3) assuming proportional odds. To answer these questions, a simulation study would be needed, which is beyond the present scope. Finally, the present work on binary outcomes might be extended to nested designs by adding a random cluster effect to models (1,2,3) and a random cluster effect per time point to model (4), as done for quantitative outcomes in [50].

## Funding

The author has nothing to report.

## Conflicts of Interest

The author declares no conflicts of interest.

## Data Availability

All data available as contingency tables in appendices D,E,F of the manuscript.

## Appendix A Proof of the Relation Between the Cumulative Logit Model and the Adjacent Categories Model for Ordinal Regression of the Change Score

The relation between the parameters of models (2) and (3) as stated in the main text can be inferred as follows:

Step 1:

$$\frac{P\left(D=1\right)}{P\left(D=-1\right)}=\frac{P\left(D=1\right)/P\left(D=0\;\text{or}-1\right)}{P\left(D=-1\right)/P\left(D=0\;\text{or}+1\right)}\;\times \;\frac{P\left(D=0\;\text{or}-1\right)}{P\left(D=0\;\text{or}+1\right)}$$

Step 2:

According to the cumulative logit model (2),

$$\frac{P\left(D=1\right)/P\left(D=0\;\text{or}-1\right)}{P\left(D=-1\right)/P\left(D=0\;\text{or}+1\right)}=\frac{\exp\left(\alpha_{0}+\alpha_{1}G\right)}{\exp\left({-\alpha }_{0}^{\prime }-\alpha_{1}^{\prime }G\right)}=\exp\left(\alpha \right),$$

where $\alpha =\left(\alpha_{0}+\alpha_{0}^{\prime }\right)+\left(\alpha_{1}+\alpha_{1}^{\prime }\right)G$ as defined in the main text.

Step 3:

Combining steps 1 and 2 gives for the cumulative logit model as result that:

$$\frac{P\left(D=1\right)}{P\left(D=-1\right)}=\exp\left(\alpha \right)\;\times \frac{P\left(D=0\;\text{or}-1\right)}{P\left(D=0\;\text{or}+1\right)}.$$

Step 4:

Combining step 3 with the adjacent categories model (3) then gives:

$$\exp\left(\delta \right)=\exp\left(\alpha \right)\;\times \frac{P\left(D=0\;\text{or}-1\right)}{P\left(D=0\;\text{or}+1\right)}$$

Now, $\frac{P\left(D=0\;\text{or}-1\right)}{P\left(D=0\;\text{or}+1\right)}<1$, and thus exp(δ)<exp(α), if and only if $\frac{P\left(D=1\right)}{P\left(D=-1\right)}>1$,

which in turn holds iff exp(δ)>1, so that 1<exp(δ)<exp(α), thus: 0<δ<α.

Likewise, $\frac{P\left(D=0\;\text{or}-1\right)}{P\left(D=0\;\text{or}+1\right)}>1$, and thus exp(δ)>exp(α), if and only if $\frac{P\left(D=1\right)}{P\left(D=-1\right)}<1$, which in turn holds iff exp(δ)<1, so that 1>exp(δ)>exp(α), thus: 0>δ>α.

In short, δ and α always have the same sign, and α is always larger in absolute size than δ, except if $\frac{P\left(D=1\right)}{P\left(D=-1\right)}=1$, in which case we have 0=δ=α, and these results hold for each of the two groups, *G* = 0 and *G* = 1.

## Appendix B Proof of the Relation Between Ordinal Regression of Change on Group With the Adjacent Categories Model and Mixed Logistic Regression for Repeated Measures

The mixed model is:

$$\mathrm{LN}\left(\frac{P\left(Y_{\mathrm{it}}=1\right)}{P\left(Y_{\mathrm{it}}=0\right)}\right)=\gamma_{0}+\gamma_{1}G+\gamma_{2}T+\gamma_{3}\mathrm{GT}+u_{i},$$

where $Y_{\mathrm{it}}$ is the measurement of person *i* at time point *t* (0 for baseline, 1 for outcome), and *G* is group (1 for treated or exposed, 0 for control), and *T* is time (0 for baseline, 1 for outcome). Finally, $u_{i}$ is a random person effect with mean 0 and variance $\sigma^{2}$. The effect of interest now is $\gamma_{3}$, the group by time interaction effect.

Define now change from baseline as: $D_{i}=Y_{i1}-Y_{i0}$, which ranges from −1 through 0 to +1, and then derive the distribution of $D_{i}$ from the mixed model as follows:

$$P\left(D_{i}=1|G\right)=P\left(Y_{i1}=1,Y_{i0}=0|G\right)=$$

$$\int \Psi \left(\gamma_{0}+\gamma_{1}G+\gamma_{2}+\gamma_{3}G+u_{i}\;\right)\left\{1-\Psi \left(\gamma_{0}+\gamma_{1}G+u_{i}\right)\right\}f\left(u\right)\mathrm{du},$$

where Ψ(.) is the logistic distribution, that is, $\Psi \left(x\;\right)=\frac{e^{x}}{1+e^{x}}$, and f(u) is the normal density with mean 0 and variance $\sigma^{2}$. Likewise,

$$P\left(D_{i}=-1|G\right)=P\left(Y_{i1}=0,Y_{i0}=1|G\right)=$$

$$\int \Psi \left(\gamma_{0}+\gamma_{1}G+u_{i}\;\right)\left\{1-\Psi \left(\gamma_{0}+\gamma_{1}G+\gamma_{2}+\gamma_{3}G+u_{i}\right)\right\}f\left(u\right)\mathrm{du}$$

From these two equations it follows, by moving the fixed part of the numerator of Ψ(.) outside the integrals for P(D=1|G) and (D=−1|G), and noting that the numerator of {1−Ψ(.)} is simply 1, that:

$$\mathrm{LN}\left(\frac{P\left(D=1|G\right)}{P\left(D=-1|G\right)}\right)=\gamma_{2}+\gamma_{3}G$$

From the equation for P(D=0|G) no such simple expression follows for $\mathrm{LN}\left(\frac{P\left(D=1|G\right)}{P\left(D=0|G\right)}\right)$, nor for $\mathrm{LN}\left(\frac{P\left(D=0|G\right)}{P\left(D=-1|G\right)}\right)$. So, the adjacent categories with proportional odds model for ordinal regression of change on group is incompatible with the mixed logistic model. Still, for $\mathrm{LN}\left(\frac{P\left(D=1|G\right)}{P\left(D=-1|G\right)}\right)$ the two are equivalent. Specifically, from the adjacent categories with proportional odds model for change, which states that.

$$\mathrm{LN}\left(\frac{P\left(D=1|G\right)}{P\left(D=0|G\right)}\right)=\delta_{0}+\delta_{1}G\;\;\text{and}\;\;\mathrm{LN}\left(\frac{P\left(D=0|G\right)}{P\left(D=-1|G\right)}\right)=\delta_{0}^{\prime }+\delta_{1}G,$$

It follows that.

$$\mathrm{LN}\left(\frac{P\left(D=1|G\right)}{P\left(D=-1|G\right)}\right)=\gamma_{2}+\gamma_{3}G,\;\text{with}\;\;\gamma_{2}=\delta_{0}+\delta_{0}^{\prime }\;\;\text{and}\;\gamma_{3}=2\delta_{1}.$$

In short, the group effect on the logodds of positive (*D* = +1) versus negative (*D* = −1) change in the adjacent categories with proportional odds model equals the group by time effect in the mixed logistic model for the repeated measures baseline (pre) and outcome (post). Their respective estimates and standard errors may differ somewhat.

If proportional odds does not hold, that is, if the group effect on $\mathrm{LN}\left(\frac{P\left(D=1|G\right)}{P\left(D=0|G\right)}\right)$, denoted by $\delta_{1}$, differs from the group effect on $\mathrm{LN}\left(\frac{P\left(D=0|G\right)}{P\left(D=-1|G\right)}\right)$, denoted by $\delta_{1}^{\prime }$, then we have $\gamma_{3}=\delta_{1}+\delta_{1}^{\prime }$. As a side note, the logodds of positive (D = +1) versus negative (D = −1) change in the control group (*G* = 0) equals $\gamma_{2}$, the simple effect of time *T*. This is in line with the McNemar test for the difference between paired samples on a binary outcome, which only considers the positive and negative differences and ignores all pairs with a zero difference.

## Appendix C Proof of the Relation Between Logistic Regression of Outcome on Group and Baseline and Mixed Logistic Regression Under Conditional Independence

As shown in Appendix B, the group by time interaction effect $\gamma_{3}$ in mixed logistic regression for repeated measures (pre, post) with a random person effect is equal to the group effect on the logodds of positive versus negative change, $\mathrm{LN}\left(\frac{P\left(D=1|G\right)}{P\left(D=-1|G\right)}\right)$, in an adjacent categories ordinal regression of change on group.

Consider now logistic regression of the outcome (posttest) on group and baseline (pretest).

$\mathrm{LN}\left(\frac{P\left(Y_{\mathrm{it}}=1\right)}{P\left(Y_{\mathrm{it}}=0\right)}\right)=\beta_{0}+\beta_{1}G_{i}+\beta_{2}Y_{i0}$, assuming absence of group*pretest interaction.

The question is: Under what conditions does $\beta_{1}=\gamma_{3}$ hold? Below, it is shown that this holds under conditional independence of group and pretest, conditional on the posttest.

Proof:

Start with the following 2 × 2 × 2 crosstable of group, pre, post, with cell entries denoting the probability of each combination (i.e., a + b + c + d + e + f + g + h = 1).

**Table:**

	Group = 0		Group = 1	
	Pre = 0	Pre = 1	Pre = 0	Pre = 1
Post = 0	a	b	e	f
Post = 1	c	d	g	h
	a + c	b + d	e + g	f + h

From Appendix B we know that:

$\mathrm{LN}\left(\frac{P\left(D=1|G\right)}{P\left(D=-1|G\right)}\right)=\gamma_{2}+\gamma_{3}G$, and so, $\mathrm{LN}\left(\frac{P\left(D=1|G=1\right)}{P\left(D=-1|G=1\right)}\right)-\mathrm{LN}\left(\frac{P\left(D=1|G=0\right)}{P\left(D=-1|G=0\right)}\right)=\gamma_{3}.$

This translates into: $\left(\frac{g/f}{c/b}\right)=\left(\frac{\mathrm{bg}}{\mathrm{cf}}\right)=\exp\left(\gamma_{3}\right)$.

From the above logistic regression of posttest on group and pretest it follows that:

$\left(\frac{g/e}{c/a}\right)=\left(\frac{h/f}{d/b}\right)=\exp\left(\beta_{1}\right)$, or equivalently, $\left(\frac{\mathrm{ga}}{\mathrm{ce}}\right)=\left(\frac{\mathrm{bh}}{\mathrm{df}}\right)=\exp\left(\beta_{1}\right).$

This equals $\exp\left(\gamma_{3}\right)$ if and only if two conditions simultaneously hold:

- $\left(\frac{\mathrm{ga}}{\mathrm{ce}}\right)=\left(\frac{\mathrm{bg}}{\mathrm{cf}}\right)\Rightarrow$ $\left(\frac{\mathrm{af}}{\mathrm{be}}\right)$ = 1, that is, independence of group and pre if post = 0,
- $\left(\frac{\mathrm{bh}}{\mathrm{df}}\right)=\left(\frac{\mathrm{bg}}{\mathrm{cf}}\right)\Rightarrow \left(\frac{\mathrm{ch}}{\mathrm{dg}}\right)=1$, that is, independence of group and pre if post = 1.

In short, the group effect in logistic regression of post on group and pre equals the group × time effect in mixed logistic regression iff group and pre are independent conditional on post.

Interestingly, as the literature on collapsibility (e.g., Greenland, Robins, & Pearl, 1999; Guo & Geng, 1995; Robinson & Jewell, 1991) shows, the group effect on the posttest adjusted for the pretest as covariate also equals the unadjusted group effect on the posttest under the above conditional independence of group and pretest.

A short proof of this is now given:

In terms of the above 2 × 2 × 2 contingency table, the unadjusted group effect on the posttest equals the adjusted group effect on the posttest iff $\left(\frac{a+b}{c+d}\right)\left(\frac{g+h}{e+f}\right)=$ $\left(\frac{\mathrm{ag}}{\mathrm{ce}}\right)$ (or $\left(\frac{\mathrm{bh}}{\mathrm{df}}\right)$ for that matter) holds under conditional independence, that is, if af=be and ch=dg both hold.

Multiplying both sides with both denominators gives:

(a+b)(g+h)(ce)=(ag)(c+d)(e+f), which can be elaborated as:

(agce+bhce+ahce+bgce)=(agce+agdf+agcf+agde)

Substituting next af=be and ch=dg and permuting letters within each term then gives:

(aceg+bdeg+adeg+bceg)=(aceg+bdeg+bceg+adeg), QED.

## Appendix D The 2 * 2 * 2 Contingency Tables of the Five Fictitious Scenarios

Scenario 1: Natural groups, parallel change (G = group, X = baseline, Y = outcome).

OR(xy.g) = 2.67; phi(xy.g) = 0.20, OR(gy.x) = 2.00, rho(gy.x) = 0.17 (for X = 0) resp. 0.15 (for X = 1).

**Table:**

	G = 0		G = 1		Total sample	
	X = 0	X = 1	X = 0	X = 1	X = 0	X = 1
Y = 0	400	100	200	100	600	200
Y = 1	300	200	300	400	600	600

Scenario 2: Natural groups, convergence (G = group, X = baseline, Y = outcome).

OR(xy.g) = 2.25, phi(xy.g) = 0.20, OR(gy.x) = 1.00, rho(gy.x) = 0.00.

**Table:**

	G = 0		G = 1		Total sample	
	X = 0	X = 1	X = 0	X = 1	X = 0	X = 1
Y = 0	360	160	240	240	600	400
Y = 1	240	240	160	360	400	600

Scenario 3: RCT, divergence (G = group, X = baseline, Y = outcome).

OR(xy.g) = 2.33, rho(xy.g) = 0.20, OR(gy.x) = 2.33, rho(gy.x) = 0.20.

**Table:**

	G = 0		G = 1		Total sample	
	X = 0	X = 1	X = 0	X = 1	X = 0	X = 1
Y = 0	350	250	250	150	600	400
Y = 1	150	250	250	350	400	600

Scenario 4: RD design, no effect (G = group, X = baseline, Y = outcome).

OR(xy.g) = 2.25, rho(xy.g) = 0.18, OR(gy.x) = 1.00, rho(gy.x) = 0.00.

**Table:**

	G = 0		G = 1		Total sample	
	X = 0	X = 1	X = 0	X = 1	X = 0	X = 1
Y = 0	420	120	180	280	600	400
Y = 1	280	180	120	420	400	600

Scenario 5: conditional independence of group and baseline, given the outcome (G = group, X = baseline, Y = outcome), *N* = 1100 instead of 1000 in G = 0 to have simple integers.

OR(xy.g) = OR(xy) = 2, rho(xy.g) = 0.17, OR(gy.x) = OR(gy) = 4, rho(gy.x) = 0.32 (if X = 0) or 0.33 (if X = 1).

OR(xg.y) = 1, OR(xg) = 1.25, rho(xy) = 0.055, near marginal independence (general: rho(xg.y) = 0 implies rho(xg) = rho(xy) × rho(gy), so, rho(xg) small then).

**Table:**

	G = 0		G = 1		Total sample	
	X = 0	X = 1	X = 0	X = 1	X = 0	X = 1
Y = 0	400	400	200	200	600	600
Y = 1	100	200	200	400	300	600

## Appendix E The 2 * 2 * 2 Contingency Tables of the Three Extra Scenarios

Scenario 6: Parallel change, with a stronger association between baseline and outcome.

OR(xy.g) = 6.00; phi(xy.g) = 0.41.

**Table:**

	G = 0		G = 1		Total sample	
	X = 0	X = 1	X = 0	X = 1	X = 0	X = 1
Y = 0	400	200	300	200	700	400
Y = 1	100	300	100	400	200	700

Scenario 7: natural groups, convergence, with a stronger association between pre and post outcome (G = group, X = baseline, Y = outcome).

OR(xy.g) = 9.00, phi(xy.g) = 0.49.

**Table:**

	G = 0		G = 1		Total sample	
	X = 0	X = 1	X = 0	X = 1	X = 0	X = 1
Y = 0	450	100	300	150	750	250
Y = 1	150	300	100	450	250	750

Scenario 8: Natural groups, almost parallel change, non‐proportional odds for the change score in the adjacent categories model.

OR(xy.g) = 2.67; phi(xy.g) = 0.16 for G = 0 respectively 0.22 for G = 1.

**Table:**

	G = 0		G = 1		Total sample	
	X = 0	X = 1	X = 0	X = 1	X = 0	X = 1
Y = 0	400	100	100	100	500	200
Y = 1	300	200	300	800	600	1000

## Appendix F The 2 * 2 * 3 * 3 Contingency Table of the Smoking Prevention Study

Smoke0, smoke1, smoke2 is current smoking status (0 = no, 1 = yes) at T0, T1, T2.

**Table:**

		Control (G = 0)			Treated (G = 1)			Total sample		
Smoke1	Smoke2	Smoke0			Smoke0			Smoke0		
		0	1	Total	0	1	Total	0	1	Total
0	0	528	31	559	638	17	655	1166	48	1214
	1	20	6	26	33	5	38	53	11	64
	Missing	46	1	47	49	1	50	95	2	97
	Total	594	38	632	720	23	743	1314	61	1475
1	0	29	7	36	14	6	20	43	13	56
	1	18	15	33	15	12	27	33	27	60
	Missing	4	1	5	2	2	4	6	3	9
	Total	51	23	74	31	20	51	82	43	125
Missing	0	36	1	37	38	1	39	74	2	76
	1	2	2	4	4	1	5	6	3	9
	Missing	33	5	38	19	4	23	52	9	61
	Total	71	8	79	61	6	67	132	14	146
Total	0	593	39	632	690	24	714	1283	63	1346
	1	40	23	63	52	18	70	92	41	133
	Missing	83	7	90	70	7	77	153	14	167
	Total	716	69	785	812	49	861	1528	118	1646
